# A Computational Journey across Nitroxide Radicals: From Structure to Spectroscopic Properties and Beyond

**DOI:** 10.3390/molecules26237404

**Published:** 2021-12-06

**Authors:** Vincenzo Barone, Marco Fusè, Sandra Mónica Vieira Pinto, Nicola Tasinato

**Affiliations:** Scuola Normale Superiore, Piazza dei Cavalieri 7, 56126 Pisa, Italy; marco.fuse@sns.it (M.F.); sandra.vieirapinto@sns.it (S.M.V.P.); nicola.tasinato@sns.it (N.T.)

**Keywords:** nitroxide radicals, geometry, IR spectra, magnetic properties, anharmonicity, large amplitude motion

## Abstract

Nitroxide radicals are characterized by a long-lived open-shell electronic ground state and are strongly sensitive to the chemical environment, thus representing ideal spin probes and spin labels for paramagnetic biomolecules and materials. However, the interpretation of spectroscopic parameters in structural and dynamic terms requires the aid of accurate quantum chemical computations. In this paper we validate a computational model rooted into double-hybrid functionals and second order vibrational perturbation theory. Then, we provide reference quantum chemical results for the structures, vibrational frequencies and other spectroscopic features of a large panel of nitroxides of current biological and/or technological interest.

## 1. Introduction

Nitroxides are one of the few families of stable organic free radicals and this feature, together with the remarkable sensitivity of their structure and spectroscopic properties to environmental effects has stimulated their widespread use as spin labels and spin probes in both biological and material chemistry [1,2,3]. The interest for this class of radicals has seen very recently a remarkable increase especially in connection with the analysis of dynamical and environmental effects by state-of-the-art computational approaches and with protein crystallography refinement [4,5,6]. Moreover, while the EPR (electronic paramagnetic resonance) spectra of a huge number of molecular systems including the NO moiety have been recorded and interpreted by means of quantum chemical (QC) computations [7,8,9,10], the situation is different concerning accurate molecular structures and, especially, vibrational spectra.

As a matter of fact, the EPR studies of spin labelled proteins can be connected with crystallographic experiments allowing the determination of a static atomistic description of the whole molecular model through structure refinement procedures [6]. The refinement requires additional a priori information, supplied in the form of chemical restraints, to compensate for the lack of high-resolution data and/or other experimental issues. Of course, the employed restraints should be at least as accurate as the sought accuracy of the refinement and can be often derived from accurate experimental data available for suitable fragments of the investigated molecular system. However, this approach is seldom exploitable for open-shell moieties, whose accurate structures are usually unknown. Under such circumstances QC computations can be effectively employed to obtain the missing information, provided that the accuracy of the selected computational model is sufficient [11]. In recent years, methods rooted in the density functional theory (DFT) have emerged as the methods of choice for medium- to large-size molecular systems not amenable to the most accurate (but also prohibitively expensive) wave-function methods. In this connection, several studies have shown that double-hybrid functionals in conjunction with partially augmented triple-zeta basis sets are particularly reliable for geometrical structures, vibrational frequencies and several other spectroscopic properties [7,12,13]. On these grounds, we have performed a comprehensive study of several prototypical nitroxide radicals using as benchmarks either available experimental data or state-of-the-art QC results. After validating the selected computational approach we provide reference data for a large panel of nitroxides of current interest for biological and/or technological applications.

## 2. Computational Details

Unless explicitly stated, calculations were performed with the Gaussian16 suite of programs [14]. The underlying electronic-structure model is rooted in the density functional theory (DFT). The hybrid B3LYP [15,16] functional in conjunction with the SNSD basis set [17] (hereafter B3) and the double hybrid B2PLYP [18] functional in conjunction with the maug-cc-pVTZ basis set [19] (where *d* functions on hydrogen atoms have been removed) (hereafter B2) were employed. Even if not explicitly indicated, Grimme’s D3 empirical dispersion corrections [20] with the Becke-Johnson damping [21] (D3BJ) were always used. In order to mimic experimental conditions, when required, bulk solvent effects were taken into account by means of the polarizable continuum model [22] within its integral equation formalism (IEF-PCM) [23]. Geometry optimizations were carried out employing very tight convergence criteria, and minima were confirmed by Hessian evaluations. Harmonic force fields were obtained using analytic energy derivatives, while higher-order derivatives were computed through numerical differentiation using a step of 0.01 $\sqrt{\mathrm{amu}}\;a_{0}$ for the displacements along the mass-weighted normal coordinates. VTP2 calculations relied on the GVPT2 approach, taking possible resonances into the proper account [11,17].

The potential energy surface (PES) ruling the large amplitude inversion motion around the nitrogen atom of the nitroxide moiety was sampled by relaxed scans and then used to solve numerically a one-dimensional Schrodinger equation by means of a quasi-variational approach rooted in the discrete variable method [24,25,26,27,28].

Isotropic hyperfine coupling constants (hcc) of free radicals observed in ESR spectroscopy are determined by the electron spin density at or near the position of any magnetic nucleus. In particular, the ${}^{14}$N hcc (the only one considered in the following and referred to as a${}_{N}$) is obtained by multiplying the corresponding spin density by 323.13 for obtaining data in MHz (115.3 for results in Gauss). Since hcc’s are particularly sensitive to both very tight and diffuse s functions, the purposely tailored EPR(III) basis set has been used for these computations [8].

Finally, in order to check the accuracy of the geometries obtained at the B2 level, the structure of the prototypical dimethylnitroxide radical (a) was optimized also at the CCSD(T)-F12/cc-pVDZ-F12 level Supplementary Materials, which is known to provide remarkably accurate results [29]. These computations were performed by the MOLPRO package [30]. In the same vein reference a${}_{N}$ values were computed at the CCSD(T) level employing the CFOUR program [31].

## 3. Results and Discussion

**Figure 1:**
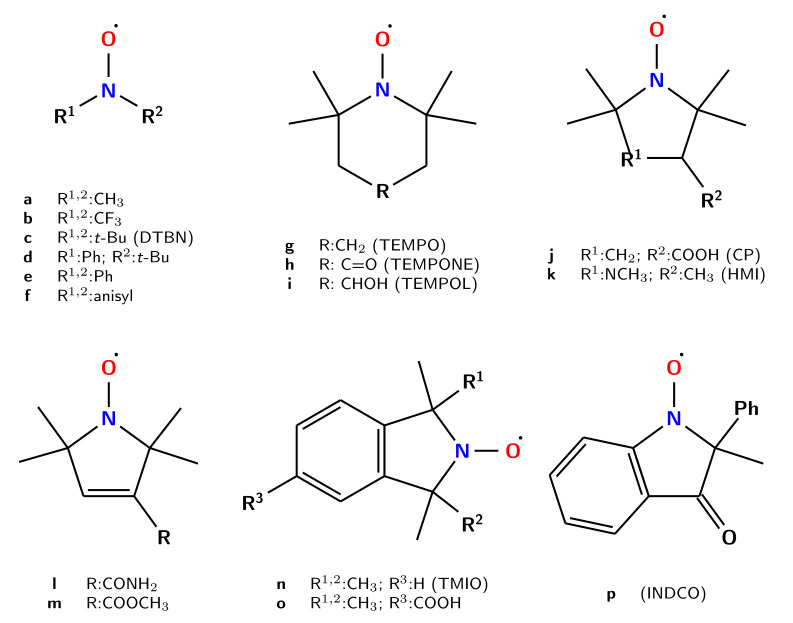
Structure and labeling of the investigated nitroxides.

The structure and labeling of the nitroxides investigated in the present paper are sketched in Figure 1. The key geometrical parameters ruling the nitroxide properties are the NO bond length (mainly affecting the magnetic g tensor and the UV-vis absorption) and the pyramidality of the nitrogen atom (mainly affecting the hyperfine tensor). Noted is that both quantities (which are not fully unrelated) are sensitive to the overall molecular backbone.

On these grounds, nitroxides can be classified in three principal groups: acyclic molecules (**a**–**f**); cyclic species containing six- (**g**–**i**) or five- (**j**–**m**) member rings, with the latter family being further split in two by the lack (**j**,**k**) or presence (**l**,**m**) of unsaturated bonds within the cycle. More complex systems containing aromatic moieties (**n**–**p**) have been also taken into account.

### 3.1. Template Molecule

It is well documented that B2 equilibrium geometries are remarkably accurate [32]; nonetheless higher level calculations performed on small model systems can provide information to correct the structures of larger molecules obtained at lower computational levels following the template molecule and/or linear regression approaches (referred to in the following as TMA and LRA, respectively) [13].

In the present connection, dimethyl nitroxide (**a**) is a suitable reference system. The geometries of the minimum and the planar transition state were optimized using the CCSD(T)-F12 ansatz [33] in conjunction with the cc-pVDZ-F12 basis set [34] (hereafter DZF12). As already mentioned, this level of theory leads to results comparable with those delivered by conventional CCSD(T) computations employing much larger basis sets [29].

In Table 1 the geometrical parameters of the **a** molecule optimized at different levels of theory are reported. Confirming previous observations for the NO bond lengths in closed-shell systems [35], despite their limited computational cost, B2 and B3 results are remarkably accurate, with the largest deviation with respect to the CCSD(T)-F12 reference being 0.003 Å. Also the CNC valence angle is well reproduced with errors below 0.3 degrees with respect to the CC reference for both B3 and B2 methods. However, the B2 method clearly outperforms the B3 counterpart for NC bond lengths, reducing the error from 0.005 to 0.001 Å. These results suggest that the geometries of larger nitroxides optimized at the B2 level can be considered sufficiently reliable, thus not requiring any ad hoc correction.

The description of the out-of-plane angle is more demanding concerning both the equilibrium value (overestimated by 3 and 2.5 degrees at the B3 and B2 level, respectively) and the height of the energy barrier to planarity (which increases from 287 to 334 and 404 cm${}^{-1}$ when going from B3 to B2 and CCSD(T)-F12 computations). It is well known that inversion motions ruled by low barriers to planarity are generally ill described by harmonic models [28], so that the effective structure does not coincide with the energy minimum even at low temperatures. Thus, this large amplitude motion (LAM) was investigated through a relaxed scan along the NO out-of-plane coordinate and it was used to solve numerically the vibrational Schrödiger equation with the help of a quasi-variational approach employing the discrete variable representation (DVR) [24,25,26,27,28].

**Figure 2:**
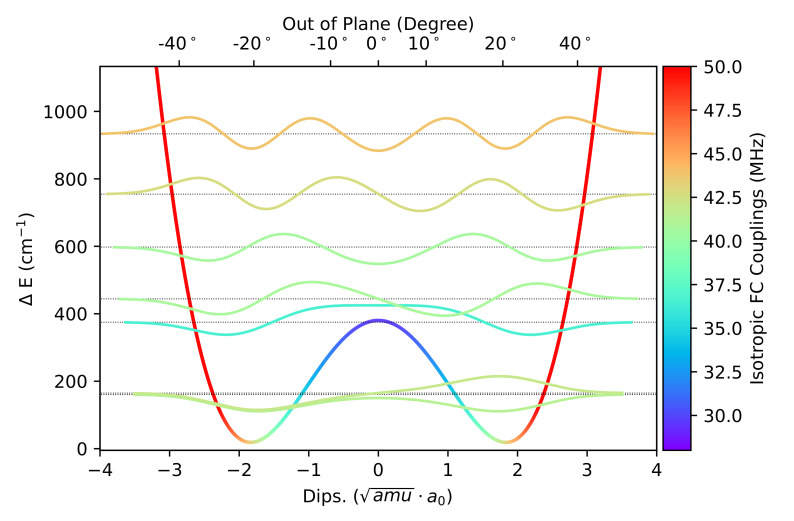
Graphical representation of the PES along the NO out-of-plane large amplitude motion computed at the B2 level. The TS energy was corrected by the values obtained at the CCSD(T)-F12/CC-PVDZ-F12 level of theory. The vibrational levels and wave functions with contribution above 0.5% at 300 K, computed using the variational DVR-based approach, are also reported. The color code reflects the value of a${}_{N}$ in MHz.

In Figure 2 the PES along the LAM is reported together with the associated vibrational levels obtained with the DVR method and the corresponding vibrational wave functions of those states relevant at 300 K (population above 0.5%). By effect of the LAM, the vibrationally averaged value of the out of plane angle decreases by about 2° with respect to the equilibrium value.

DFT methods are known to underestimate the absolute values of a${}_{N}$ in nitroxide systems [4]. On these grounds, the performance of the double hybrid B2PLYP functional in conjunction with the purposely tailored EPR(III) basis set was evaluated for the Me${}_{2}$NO${}^{\cdot }$ model system taking the results obtained at the CCSD(T) level (hereafter CC) as references. The results collected in Table 2 show that the B3 results consistently underestimate the CC reference values (by about 5 MHz) and that the B2 method represents a remarkable improvement leading to quantitative agreement with CC for the pyramidal equilibrium structure and a slight underestimation (about 1 MHz) for the planar transition state. This trend is not surprising since for planar structures a${}_{N}$ is determined only by spin-polarization (which is particularly hard to be described due to spin contamination and role of exact exchange). As a matter of fact, increasing the contribution of Hartree-Fock exchange (cfr. B3 and BHLYP results in Table 2) leads to better results, which are further improved by inclusion of a fraction of second order many-body perturbation (B2 results).

**Figure 3:**
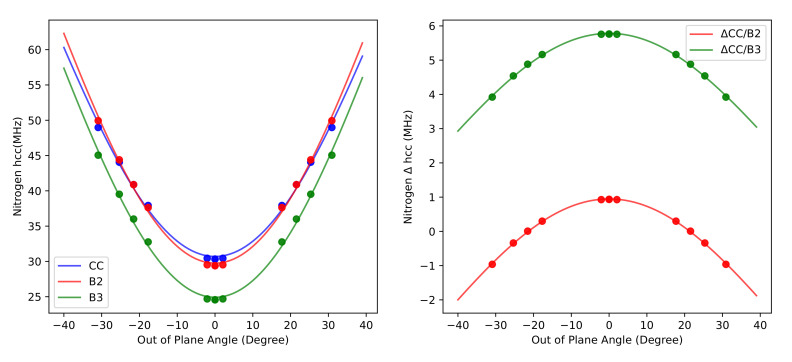
a${}_{N}$ profile as a function of the out of plane angle at different levels of theory in conjunction with the EPR(III) basis set. The curves were fitted with $\Delta \theta =a\cdot cos^{2}\left(\theta \right)+b$ functions.

Furthermore, Figure 3 shows that the CC, B2, and B3 results as a function of θ (the angle formed by the NO bond with the CNC plane) are quantitatively fitted by the function

(1) $$\Delta \theta =a\cdot cos^{2}\left(\theta \right)+b$$

with a=−71.65,−78.78,−78.53 and b=102.35,108.53,103.47 MHz for CC, B2, and B3 methods, respectively.

This is exactly the expected behaviour since θ drives the increase of the contribution of nitrogen s orbitals to the π SOMO (singly occupied molecular orbital) of nitroxides.

These results can be employed to correct the a${}_{N}$ for larger nitroxides by a composite scheme in which the difference between CC and B2 a${}_{N}$ is estimated by the respective values issuing from Equation (1) at a θ angle equal to that of the target molecule.

Finally, vibrational averaging along the inversion LAM decreases the value of a${}_{N}$ at 300 K by 1.3, 1.6 and 2.5 MHz at the B3, B2 and CC level of theory, respectively. The corresponding contribution obtained from the perturbative VPT2 approach is 1.8 MHz, showing a remarkable agreement with the variational counterpart.

### 3.2. Geometries

The geometrical parameters describing the C${}_{1}$C${}_{2}$NO moiety of all the nitroxides shown in Figure 1 computed at the B2 level are collected in Table 3.

In the case of the heterogeneous class of acyclic molecules, the geometrical parameters strongly depend on the nature of the substituent. It is quite apparent that bulky substituents decrease the pyramidality of the nitrogen atom leading to nearly-planar structures, which become exactly planar in the presence of aryl substituents due to more effective delocalization of π electrons.

As already pointed out in previous studies [9,36,37], a nearly planar geometry of the NO group is generally observed when it is included in a five-member ring, which becomes pyramidal when included in a six-member ring. All the six-member ring molecules share the piperdine-N-oxyl scaffold, which usually adopts a chair-like structure with the NO group occupying an equatorial position. In the saturated five-member ring systems, only small deviations from the planarity of the NO nitrogen are observed, with the ring displaying a twisted structure with the atoms opposed to the nitrogen lying outside the mean plane of the system. Resembling the case of the aryl substituents, in the acyclic system the presence of an aromatic ring forces the planarity of the nitroxides. Nonetheless, when the substituents on the double bond are not strongly conjugated, as in the case of **l**, small distortions of the rings are observed. In terms of Cremer and Pople coordinates [38], the total puckering amplitude is small (below 0.1 Å) if compared to the values generally observed in other systems containing five member rings (usually between 0.2 and 0.35 Å) [35]. Although this leads to an out-of-plane angle of about 10° in the most stable structure, a scan of the potential energy surface along the out-of-plane coordinate revealed that the planar structure is easily accessible even at very low temperatures. Noted is that that two equivalent minima with opposite signs of the NO out-of-plane angle exist and that their interconversion requires also the mirror conformation of the side chain.

The NO bond length shows little variability for all cyclic nitroxides, with its value being shorter by about 0.01 Å for five-member rings. With the exception of **b**, which bears two strongly withdrawing CF${}_{3}$ groups, acyclic nitroxides have longer NO bond lengths ranging from 1.280 to 1.286 Å.

### 3.3. NO Stretching Frequencies

Despite the ubiquitous presence of the NO group in radical traps and EPR spin probes, little attention has been paid to its vibrational characterization. At variance with other functional groups like (e.g., CO or CN), the infrared (IR) intensity of the NO stretching is generally low and its identification is not always straightforward, thus leading to inconsistent assignments [39]. Recently, Rintoul and coworker [39] reviewed the available experimental vibrational spectra of nitroxides and performed a comprehensive comparison with the results issuing from DFT calculations. A scaling factor of 0.976 was applied to DFT results in order to account for systematic errors related to the limits of the computational model and/or to the underlying harmonic approximation. Aided by the computational results, the authors found several incorrect band assignments and proposed the range between 1340 and 1450 cm${}^{-1}$ for the $\nu_{NO}$ vibration.

Within the set of nitroxide radicals collected in ref. [39] for which experimental results are available, we selected five acyclic nitroxides, together with seven cyclic radicals (four five-member and three six-emember rings), whose experimental data are sufficiently reliable. When the experimental spectra were recorded in solution, bulk solvent effects were taken into account by mean of the polarizable continuum model [23]; nonetheless, most of the experimental values were recorded in KBr or Nujol, whose perturbing effect was neglected in the computations. The NO stretching frequencies computed at B3 and B2 levels for all the nitroxides sketched in Figure 1 are compared to the available experimental data in Table 4.

It is well known that harmonic calculations overestimate the vibrational frequencies, so that empirical scaling factors are usually employed to improve the agreement with experiment. When the dimension of the system does not allow full anharmonic calculations by high-level methods, an effective alternative not involving any empirical factor is offered by the so-called hybrid approaches in which harmonic frequencies at a high level of theory (here B2) are coupled to anharmonic corrections evaluated at a lower level (here B3) leading to $\nu_{H}=\omega_{B2}+\left(\nu_{B3}-\omega_{B3}\right)$. The reliability of the issuing B2/B3 hybrid scheme is well documented [11,12].

**Figure 4:**
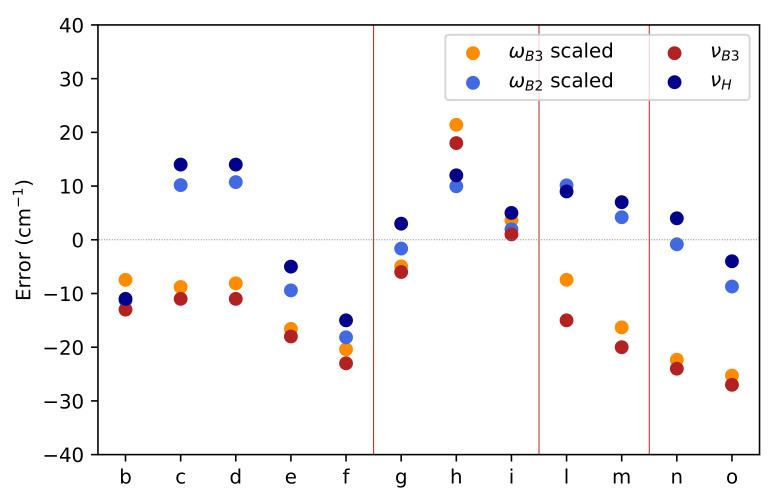
Comparison between experimental frequencies of NO stretchings and the counterparts issued from scaled harmonic (B3 in orange and B2 in light blue) and anharmonic (B3 in red and hybrid in dark blue) frequencies. The scaling factors are 0.976 and 0.980 for $\omega_{B3}$ and $\omega_{B2}$, respectively. Vertical lines separate the different nitroxide families defined in the main text.

In Figure 4 the relative errors of the computed NO stretching frequencies with respect to the experimental counterparts are plotted for the different families of nitroxide radicals defined above. Even the cheap B3 level of theory performs adequately in the case of acyclic and six-member ring molecules, with comparable values of scaled B3 and B2 harmonic frequencies. However, the agreement between the two levels of theory decreases for nitroxides involving five-member rings (**l**,**m**,**n**,**o**) with a significant overestimation of $\nu_{NO}$ at the B3 level. As a matter of fact, the mean error decreases from 21 (B3) to 6 cm${}^{-1}$ (B2), with the last value representing an excellent achievement when taking into account the variability of the experimental conditions. For all the considered nitroxides the anharmonic corrections are very close (the range being 29–34 cm${}^{-1}$) so that a constant correction of 30 cm${}^{-1}$ performs a remarkable job. Alternatively, the computational burden can be reduced by carrying out reduced dimensionality computations in which anharmonic contributions are computed only for normal modes strongly coupled to the NO stretching.

Since a reliable estimate of the coupling is provided by the ratio between the two-mode cubic constants involving the NO mode ($K_{iij}$, which can be obtained by just two additional Hessian evaluations) and the harmonic frequency of the coupled mode ($\omega_{i}$), a suitable reduced dimensionality scheme can be selected a priori in terms of a pre-defined threshold. The results collected in Table 5 for representative nitroxides show that a threshold of 0.03 leads to very accurate results by the inclusion of at most 10 modes together with the NO stretching.

A final comment is in order about the correlation between the vibrational frequency of the NO stretching and suitable geometrical parameters (e.g., NO bond length or pyramidality of the nitrogen environment). Unfortunately, several attempts to derive sufficiently accurate relationships provided disappointing results possibly because of the delocalization of the NO stretching and the ensuing involvement of several geometrical parameters.

### 3.4. Hyperfine Coupling Constants

The isotropic hyperfine couplings of free radicals are primarily ruled by the shape of the SOMO [9]. For instance, the hcc’s of the central atom of π radicals (nitrogen in the case of nitroxides) should vanish for planar structures because of the corresponding lack of any contribution of its s orbitals to the SOMO. The only remaining contribution to a${}_{N}$ is spin polarization. As a consequence, as shown by the results collected in Table 6, the a${}_{N}$ of planar or nearly planar nitroxides are smaller than those of the pyramidal counterparts. As already mentioned, the correct reproduction of spin polarization is particularly difficult at the DFT level with both high percentages of Hartree-Fock exchange and second order perturbative contributions playing a role to obtain improved results. If this interpretation is correct, the error of DFT results should always decrease with the pyramidality at the radical center due to the lower relative contribution of spin-polarization to the overall a${}_{N}$. The $cos^{2}\left(\theta \right)$ dependence of the error discussed in detail in the section devoted to the template dimethylnitroxide molecule is a direct consequence of this model. On these grounds, two strategies can be employed to improve the computed a${}_{N}$ values. In the first one, closely resembling the ONIOM approach [40], the a${}_{N}$ computed for the target molecule by a low-level method (here B2) is corrected by the difference of the a${}_{N}$ computed for a template molecule (TM, here dimethylnitroxide) at the geometry of the target molecule employing high (here CC) and low level methods. This approach (hereafter referred to as ΔTM) requires a new CC computation of the model system for each target molecule. However, the discussion above suggests an even simpler approach (hereafter referred to as Δθ) not requiring any additional computation at the CC level. In fact, if the dominant contribution to the error is related to the value of the out-of-plane angle, it is sufficient to fit once for ever the parameters of Equation (1) at the CC level and then use this Equation and the specific value of the out of plane angle for computing the correction to be applied to the B2 value.

The results collected in Table 6 show that the simplified model provides remarkably accurate results, thus allowing to correct the computed a${}_{N}$ of any nitroxide simply in terms of its out of plane angle. In spite of non negligible differences between the CNC angle of the real molecule and that of the dimethylnitroxide model at the same out of plane angle, the discrepancies are always smaller than 0.3 MHz, a value well within the expected error bar of even the most accurate QC models. We have thus at our disposal an effective strategy for computing equilibrium a${}_{N}$ values, which can be extended to other density functionals without any additional CC computation, but simply fitting the a and b parameters of Equation (1) for the new functional (by means of cheap computations for the dimethylnitroxide template molecule) to be compared with the already available CC counterparts.

Experimental results actually refer to vibrationally averaged quantities at the temperature of the experiment. These contribution can be effectively obtained in the framework of the GVPT2 model since cubic and semi-diagonal quartic force constants can be computed with sufficient accuracy employing denstity functionals cheaper than double hybrids. In view of previous experience [41], we have employed the B3 model, whose results are also collected in Table 6. It is apparent that the effect of vibrational averaging is particularly significant for some planar or nearly-planar systems, but it cannot be neglected also in other cases in order to obtain quantitative results.

The accuracy of the results collected in Table 6 can be guessed by comparison with the experimental data for the large and particularly demanding INDCO radical **p**. Summing the contributions of columns 5,6 and 8 we arrive to a${}_{N}$ = 25.6 MHz and bulk solvent effects evaluated at the PCM level [22] for the benzene solvent employed in experiments add further 0.5 MHz leading to a final estimate of 26.1 MHz, in remarkable agreement with the experimental value of 25.9 MHz [42].

## 4. Concluding Remarks

In the present paper we have proposed and validated a general and robust strategy providing accurate structures, vibrational and magnetic properties of nitroxide radicals by means of hybrid and double-hybrid functionals in conjunction with suitable basis sets. Anharmonicities and vibrational averaging effects on different properties can be taken into account employing the effective generalized vibrational perturbation theory to the second order. The geometrical parameters and vibrational frequencies obtained at this level are remarkably accurate, whereas magnetic properties can be further improved by means of coupled cluster computations performed for a template molecule [8].

However, environmental effects cannot be neglected for studies in condensed phases. For innocent solvents (or more generally when inter-molecular hydrogen bonds are not present) polarizable continuum models can be profitably used and their most effective implementations do not add any significant computational burden [22]. The situation is different for hydrogen-bonding environments since the geometry and magnetic properties of the NO moiety are strongly affected by this kind of interactions. Several studies have been devoted to this problem [4,5,9,10], but a systematic investigation for a large panel of nitroxides and solvents is still lacking. Work is in progress in our laboratory to extend the strategy reported above to those situations by means of effective variational-perturbative approaches resembling those already applied with remarkable success for other spectroscopic properties [43].

## Figures and Tables

**Table 1:** Geometrical parameters of the dimethylnitroxide template molecule at different levels of theory.

		Minimum				Transition State			
Level of Theory	Basis Sets	rNO	rCN	CNC	CCNO	rNO	rCN	CNC	CCNO
B3	SNSD	1.2798	1.4593	118.6	153.9	1.2804	1.4545	119.4	180.0
B2	maugTZ	1.2796	1.4549	118.7	153.3	1.2802	1.4504	119.3	180.0
CCSD(T)-F12	DZ-F12	1.2773	1.4554	118.3	150.8	1.2774	1.4491	119.6	180.0

**Table 2:** a${}_{N}$ (in MHz) for the dimethylnitroxide template molecule computed at B2 geometries employing different QC approaches.

Me${}_{2}$NO${}^{\cdot }$			
**Level of Theory**	**Basis Sets**	**Minimum**	**TS**
B3	EPR(III)	39.38	24.46
BHLYP	EPR(III)	43.63	31.54
B2	EPR(III)	44.21	29.26
CCSD(T)	EPR(III)	44.26	30.20

**Table 3:** Geometric parameters of the selected molecules at the B2 level of theory.

Mol	NO	C${}_{1}$N	C${}_{2}$N	C${}_{1}$NC${}_{2}$	C${}_{1}$NO	C${}_{2}$NO	C${}_{1}$C${}_{2}$NO
**a**	1.280	1.455		118.70			153.30
**b**	1.267	1.460	1.470	120.98	117.37	120.18	165.77
**c**	1.284	1.507	1.506	127.26	116.18	113.10	158.02
**d**	1.282	1.503	1.420	125.17	117.79	117.01	177.76
**e**	1.283	1.421		123.13	118.43		180.00
**f**	1.286	1.418		123.06	118.47		180.00
**g**	1.282	1.495		124.11	115.81		155.75
**h**	1.280	1.496		124.06	115.62		154.56
**i**	1.281	1.494		123.97	115.87		155.74
**j**	1.271	1.481	1.483	115.55	121.88	122.50	176.97
**k** ${}^{a}$	1.271	1.476	1.475	113.90	122.43	123.39	174.11
**l**	1.270	1.487	1.481	114.47	122.54	122.14	169.69
**m**	1.270	1.486	1.480	114.84	122.71	122.45	180.00
**n**	1.270	1.485	1.485	115.63	122.22	122.15	180.000
**o**	1.271	1.485	1.485	115.59	122.21	122.21	180.00
**p**	1.270	1.484	1.389	111.71	123.68	124.57	177.83

${}^{a}$ Only the most stable (*R*,*S*) configuration is reported.

**Table 4:** Experimental vs. computed vibrational frequencies (cm${}^{-1}$) for the NO stretching of different nitroxides labelled according to Figure 1.

Mol.	$\nu_{\exp}$	$\omega_{B3}$	$\nu_{B3}$	$\Delta_{B3}^{\mathrm{anh}}$	$\omega_{B2}$	$\nu_{H}$ ${}^{f}$
**b**	1397 ${}^{a}$	1439	1410	29	1437	1408
**c**	1342 ${}^{b}$	1384	1350	34	1359	1325
**d**	1370 ${}^{b}$	1412	1379	33	1387	1354
**e**	1342 ${}^{c}$	1392	1360	32	1379	1347
**f**	1346 ${}^{c}$	1400	1369	31	1392	1361
**g**	1339 ${}^{c}$	1377	1345	31	1368	1336
**h**	1380 ${}^{d}$	1392	1362	30	1398	1367
**i**	1371 ${}^{d}$	1401	1370	31	1397	1366
**j**	-	1472	1440	32	1453	1421
**k**	-	1485	1456	29	1461	1432
**l**	1438 *^e^*	1481	1453	28	1457	1429
**m**	1435 *^e^*	1487	1455	32	1460	1427
**n**	1428 *^e^*	1486	1452	34	1458	1424
**o**	1427 ${}^{c}$	1488	1454	34	1465	1431
**p**	-	1468	1438	30	1456	1426

${}^{a}$ Gas-phase; ${}^{b}$ KBr; ${}^{c}$ tBuOH solution; ${}^{d}$ CCl${}_{4}$ solution; ^e^ Nujol; ${}^{f}\nu_{H}=\omega_{B2}-\Delta_{B3}$.

**Table 5:** Reduced dimensionality results obtained employing a $K_{iij}/\omega_{i}$ threshold of 0.03.

Mol	$\Delta_{B3}^{\mathrm{full}}$	$\Delta_{B3}^{\mathrm{red}}$	Modes
**b**	29	29	7
**d**	33	35	11
**e**	32	33	11
**f**	31	32	7
**h**	30	30	8
**i**	30	35	7

**Table 6:** Equilibrium NO bond length (Å), out of plane angle θ (in degrees) and a${}_{N}$ (in MHz) computed at the B2 level together with high-level corrections from the template molecule model (ΔTM in MHz) and B3 vibrational corrections at 300 K ($\Delta_{vib}$ in MHz). In the last column, the correction obtained by Equation (1) (Δθ in MHz) is reported. The column $\Delta_{CNC}$ reports the difference between the CNC angle of the real system and that of the dimethylnitroxide model (**a**) at the same θ angle.

Mol	NO	$\Delta_{\mathrm{CNC}}$	θ	a${}_{N}$	$\Delta_{\mathrm{vib}}$	ΔTM	Δθ ${}^{a}$
**b**	1.267	1.4	12.26	23.12	2.71	0.68	0.63
**c**	1.284	8.0	20.13	40.14	1.66	0.44	0.10
**d**	1.282	5.6	2.00	25.54	2.62	1.05	0.94
**e**	1.283	3.6	0.00	20.11	1.10	0.99	0.95
**f**	1.286	3.5	0.00	21.10	2.73	0.99	0.95
**g**	1.282	5.1	21.70	40.42	1.14	0.22	−0.03
**h**	1.280	5.2	22.79	40.62	2.07	0.09	−0.12
**i**	1.281	4.9	21.70	40.18	1.34	0.20	−0.03
**j**	1.271	−4.0	2.60	30.82	6.23	1.03	0.93
**k**	1.271	−5.7	4.97	31.21	7.17	0.99	0.89
**l**	1.270	−5.1	8.68	32.83	6.22	0.82	0.78
**m**	1.270	−4.7	0.00	30.66	5.21	0.98	0.95
**n**	1.271	−3.9	0.00	30.64	2.70	0.98	0.95
**o**	1.270	−3.9	0.00	30.45	5.47	0.99	0.95
**p**	1.270	−7.8	1.80	20.27	4.40	0.98	0.94

${}^{a}$Δθ = 7.13 × cos${}^{2}(\theta$) − 6.18.

## Data Availability

Data is contained within the article or Supplementary Materials.

## Supplementary Materials

The following material are available online: Cartesian coordinates in xyz format at B2 level of theory of all the investigated molecules but **a**; Cartesian coordinates in xyz format at CCSD(T) level of theory of **a**.
