# Supplementary material for: A Computational Journey across Nitroxide Radicals: From Structure to Spectroscopic Properties and Beyond
Source: Molecules. 2021 Dec 6;26(23):7404. doi: 10.3390/molecules26237404 (PMC8659111; doi:10.3390/molecules26237404)
Supplement: Supplementary file 1 [file molecules-26-07404-s001.zip › molecules-1474141-supplementary.pdf]

Supporting Information: A computational journey across nitroxide radicals: from structure to spectroscopic properties and beyond

Vincenzo Barone,* Marco Fusè, Sandra Mónica Vieira Pinto, and Nicola Tasinato

Scuola Normale Superiore, Piazza dei Cavalieri 7, 56126 Pisa, Italy

E-mail: vincenzo.barone@sns.it

Contents

1 Cartesian Coordinates **S-3**

1.1 CCF12 Geometry S-4

1.2 B2 Geometry S-5

1 Cartesian Coordinates

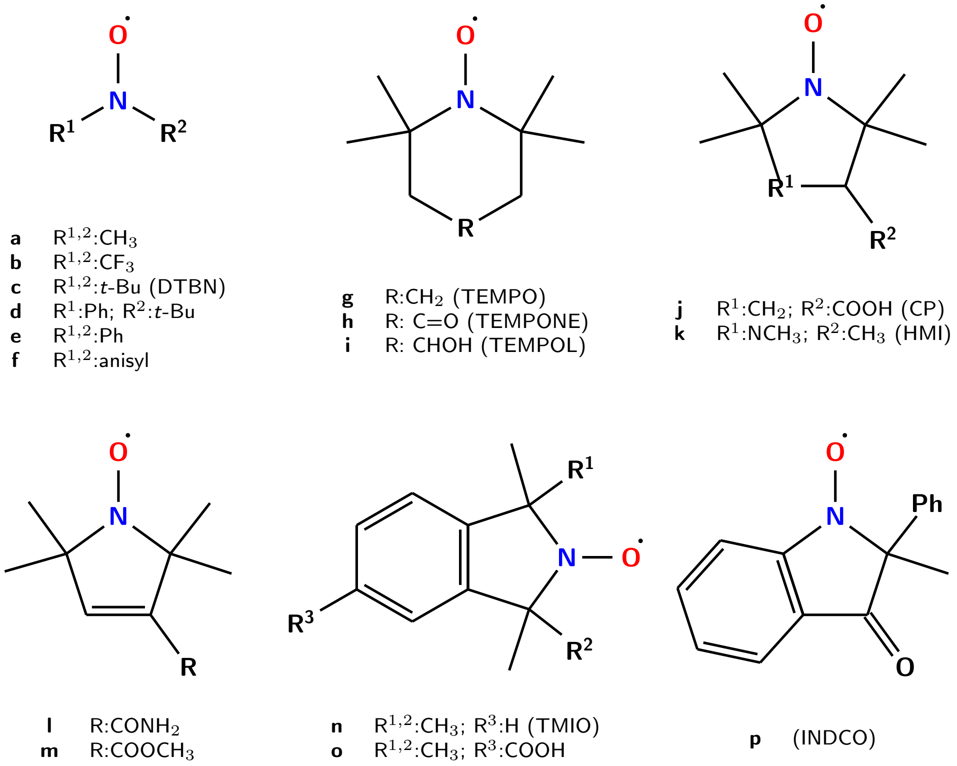

Figure S1: Molecular structures of the systems under investigation.

1.1 CCF12 Geometry

Here below the Cartesian coordinates of the dimethylnitroxide at the CCSD(T)-F12/CC-PVDZ-F12 level of theory are reported.

a-min-f12

```
  10
CCSD(T)-F12/CC-PVDZ-F12  ENERGY=-209.43744669
N         -0.1652567057       -0.0410695080        0.0000070584
O          0.0495014046       -1.3002380842       -0.0001787706
C          0.0403166370        0.6763798830       -1.2481398981
C          0.0403183822        0.6760979646        1.2483157350
H         -0.5040360628        1.6199617649       -1.2231184679
H         -0.5040343525        1.6196854736        1.2235081791
H         -0.3268452931        0.0488191718       -2.0553214283
H         -0.3268424194        0.0483549635        2.0553560203
H          1.1057957938        0.8783604365       -1.4008125401
H          1.1057977525        0.8780440313        1.4010325017
```

a-TS-f12

```
  10
CCSD(T)-F12/CC-PVTZ-F12  ENERGY=-209.46760885
N         -0.0000780709       -0.0420872872       -0.0000001760
O         -0.0001097767       -1.3184700435       -0.0000021383
C          0.0000052975        0.6864650274       -1.2519555359
C          0.0000053076        0.6864657482        1.2519558450
H         -0.8879137679        1.3204891671       -1.3246093784
H         -0.8879137226        1.3204906140        1.3246085141
H          0.0000538533       -0.0454683744       -2.0535345932
H          0.0000538291       -0.0454663814        2.0535367343
H          0.8879422478        1.3204796896       -1.3244875003
H          0.8879422075        1.3204811607        1.3244866017
```

1.2 B2 Geometry

b

```
10
Enegy:    -805.03575048
N              -0.006524        0.615139       -0.117025
O              -0.052767        1.878762       -0.032136
C              -1.268928       -0.110997       -0.014382
C               1.280615       -0.086148       -0.008236
F              -2.134228        0.396584       -0.874637
F              -1.773100       -0.001488        1.211208
F              -1.065361       -1.395002       -0.285701
F               1.290333       -0.870564        1.070367
F               1.485688       -0.847361       -1.079704
F               2.238521        0.807209        0.086065
```

c

```
28
Enegy:    -445.37671366
N              -0.003065       -0.489337       -0.187150
O               0.033998       -1.768600       -0.081257
C              -1.354020        0.153614       -0.009090
C               1.345514        0.157785       -0.009617
C              -2.403678       -0.937650       -0.225251
H              -2.311327       -1.732371        0.506729
H              -3.389151       -0.482497       -0.136654
H              -2.309534       -1.379407       -1.214415
C              -1.499941        0.703535        1.414584
H              -0.810080        1.518178        1.618927
H              -2.511962        1.084226        1.551758
H              -1.331583       -0.086731        2.143967
C              -1.594073        1.250033       -1.054175
H              -1.337503        0.891160       -2.049682
H              -2.652914        1.505111       -1.052214
H              -1.040779        2.160708       -0.857845
C               1.363689        1.658324       -0.286507
H               2.385249        2.005762       -0.138453
H               1.084443        1.884814       -1.312251
H               0.729989        2.225710        0.388791
C               1.824978       -0.116443        1.421250
H               1.769912       -1.181542        1.630640
H               2.858825        0.209697        1.530444
H               1.223150        0.417437        2.153915
C               2.283728       -0.526618       -1.008400
H               3.284530       -0.108451       -0.908980
H               2.324004       -1.595316       -0.826803
H               1.940230       -0.361195       -2.028847
```

d

26
Enegy: -519.14908212

O	-0.748000	-0.922176	1.774595
N	-0.297771	-0.678538	0.599170
C	-0.131808	0.685789	0.242165
C	0.105023	1.598680	1.274458
C	0.255292	2.946511	0.989315
C	0.164297	3.407984	-0.321087
C	-0.087951	2.502453	-1.343994
C	-0.239282	1.147991	-1.071459
H	0.161863	1.229017	2.285603
H	0.446285	3.640090	1.795817
H	-0.181724	2.847829	-2.363759
H	-0.466519	0.473982	-1.878970
H	0.280535	4.459357	-0.539961
C	0.056552	-1.850438	-0.272046
C	-1.124684	-2.167985	-1.195744
C	1.341782	-1.569178	-1.053957
C	0.302440	-3.044079	0.650912
H	-2.023883	-2.330658	-0.604456
H	-1.323354	-1.372154	-1.908773
H	-0.913730	-3.077161	-1.757891
H	2.145608	-1.286372	-0.375918
H	1.639038	-2.480310	-1.570648
H	1.234098	-0.786109	-1.796488
H	0.594992	-3.895022	0.037812
H	1.101208	-2.831697	1.358067
H	-0.587810	-3.301422	1.214174

e

24			
Enegy:	-592.92786581		
N	-0.000000	0.000000	0.978789
O	-0.000000	0.000000	2.261650
C	-0.000000	1.249611	0.302168
C	0.600682	2.339838	0.930101
C	-0.633795	1.404680	-0.931032
C	0.584016	3.579978	0.308628
H	1.061562	2.196072	1.894509
C	-0.644638	2.652634	-1.540460
H	-1.128866	0.566642	-1.396635
C	-0.033218	3.742174	-0.928679
H	1.056053	4.422845	0.792652
H	-1.143660	2.773553	-2.491213
H	-0.044046	4.710146	-1.408079
C	-0.000000	-1.249611	0.302168
C	0.633795	-1.404680	-0.931032
C	-0.600682	-2.339838	0.930101
C	0.644638	-2.652634	-1.540460
H	1.128866	-0.566642	-1.396635
C	-0.584016	-3.579978	0.308628
H	-1.061562	-2.196072	1.894509
C	0.033218	-3.742174	-0.928679
H	1.143660	-2.773553	-2.491213
H	-1.056053	-4.422845	0.792652
H	0.044046	-4.710146	-1.408079

f

32
Enegy: -821.89861748

O	-0.000001	2.832909	-0.000001
N	-0.000001	1.547215	-0.000005
C	1.246127	0.871140	0.039924
C	2.325062	1.499172	0.669162
C	3.561961	0.887643	0.694556
C	3.749907	-0.357873	0.087018
C	2.679319	-0.978794	-0.554856
C	1.432655	-0.360378	-0.578519
O	5.004311	-0.876689	0.170804
C	5.240909	-2.133006	-0.445831
C	-1.246127	0.871139	-0.039931
C	-2.325069	1.499176	-0.669154
C	-1.432650	-0.360385	0.578503
C	-3.561967	0.887647	-0.694542
C	-2.679314	-0.978800	0.554845
C	-3.749907	-0.357873	-0.087015
O	-5.004312	-0.876689	-0.170793
C	-5.240904	-2.133012	0.445833
H	-2.171509	2.466148	-1.121619
H	-4.403244	1.357397	-1.182446
H	-0.615429	-0.835645	1.098934
H	-2.799650	-1.928956	1.049613
H	-5.060595	-2.085502	1.520837
H	-6.285209	-2.361896	0.264074
H	-4.615783	-2.912328	0.006992
H	2.171499	2.466140	1.121634
H	4.403232	1.357388	1.182471
H	2.799658	-1.928945	-1.049631
H	0.615439	-0.835634	-1.098961
H	4.615782	-2.912327	-0.007003
H	5.060610	-2.085486	-1.520836
H	6.285212	-2.361892	-0.264063

g

29
Enegy: -483.47268394

N	0.203718	-0.715521	-0.000000
O	0.063008	-1.989504	-0.000000
C	0.017709	-0.040268	1.320439
C	0.487681	1.415451	1.237575
C	-0.014961	2.143633	0.000000
C	0.487681	1.415451	-1.237575
C	0.017709	-0.040268	-1.320439
C	-1.458434	-0.131601	1.728859
H	-1.793536	-1.163874	1.657562
H	-1.572783	0.201645	2.759950
H	-2.099171	0.484953	1.103245
C	0.870582	-0.793926	2.339386
H	0.808493	-0.287579	3.302134
H	0.526381	-1.817041	2.453174
H	1.912574	-0.814182	2.024226
H	0.170628	1.921478	2.150388
H	1.580238	1.429945	1.230632
H	-1.104106	2.203037	0.000000
H	0.349797	3.171048	0.000000
H	0.170628	1.921478	-2.150388
H	1.580238	1.429945	-1.230632
C	-1.458434	-0.131601	-1.728859
H	-1.572783	0.201645	-2.759950
H	-1.793536	-1.163874	-1.657562
H	-2.099171	0.484953	-1.103245
C	0.870582	-0.793926	-2.339386
H	0.526381	-1.817041	-2.453174
H	0.808493	-0.287579	-3.302134
H	1.912574	-0.814182	-2.024226

h

```
28
Enegy:    -557.48595647
N                 0.000000         -0.000000         -1.035245
O                 0.000000         -0.000000         -2.314394
C                -0.809371          1.032704         -0.334346
C                -1.165270          0.531319          1.068538
C                -0.000000          0.000000          1.870396
C                 1.165270         -0.531319          1.068538
C                 0.809371         -1.032704         -0.334346
C                 0.000000          2.334254         -0.273205
H                 0.306090          2.618521         -1.277680
H                -0.609973          3.134281          0.145048
H                 0.891603          2.232980          0.343084
C                -2.093764          1.262163         -1.127473
H                -2.720605          1.975472         -0.593976
H                -1.872649          1.652015         -2.115640
H                -2.645250          0.330865         -1.243621
H                -1.888566         -0.284314          0.988300
H                -1.647916          1.321468          1.641041
O                -0.000000          0.000000          3.083857
H                 1.647916         -1.321468          1.641041
H                 1.888566          0.284314          0.988300
C                -0.000000         -2.334254         -0.273205
H                -0.306090         -2.618521         -1.277680
H                 0.609973         -3.134281          0.145048
H                -0.891603         -2.232980          0.343084
C                 2.093764         -1.262163         -1.127473
H                 2.720605         -1.975472         -0.593976
H                 1.872649         -1.652015         -2.115640
H                 2.645250         -0.330865         -1.243621
```

i

```
30
Enegy:    -558.67900583
C              -0.211615        1.806288        0.000000
C               0.349220        1.123398        1.234515
C               0.015232       -0.368896        1.319216
C              -1.446196       -0.595469        1.729503
C               0.933914       -1.037734        2.340282
H              -1.299289        1.754209        0.000000
H               1.021881        3.324689        0.000000
H               1.437005        1.243442        1.222048
H              -0.020043        1.615952        2.133867
H              -1.591553       -0.264492        2.757149
H              -1.680135       -1.655869        1.668602
H              -2.144294       -0.049644        1.099788
H               1.973669       -0.961406        2.026511
H               0.685511       -2.088350        2.452893
H               0.822978       -0.539442        3.302568
N               0.265133       -1.024742       -0.000000
O               0.066055        3.205850        0.000000
O               0.246300       -2.305775       -0.000000
C               0.349220        1.123398       -1.234515
C               0.015232       -0.368896       -1.319216
H               1.437005        1.243442       -1.222048
H              -0.020043        1.615952       -2.133867
C              -1.446196       -0.595469       -1.729503
C               0.933914       -1.037734       -2.340282
H              -1.591553       -0.264492       -2.757149
H              -1.680135       -1.655869       -1.668602
H              -2.144294       -0.049644       -1.099788
H               1.973669       -0.961406       -2.026511
H               0.685511       -2.088350       -2.452893
H               0.822978       -0.539442       -3.302568
```

j

```
29
Enegy:   -632.71898619
N             1.275100      0.564405     -0.027376
O             2.202555      1.429266      0.063969
C             1.530220     -0.891897      0.082998
C             0.104818     -1.438963      0.219346
C            -0.772433     -0.423322     -0.520277
C            -0.145668      0.954155     -0.181657
C             2.225885     -1.367903     -1.194084
H             3.144311     -0.803714     -1.343095
H             2.473742     -2.425942     -1.115836
H             1.591973     -1.226330     -2.068552
C             2.399094     -1.169946      1.302180
H             2.559915     -2.242290      1.407025
H             3.363188     -0.678279      1.195951
H             1.922699     -0.799200      2.208264
H            -0.179649     -1.485762      1.267823
C            -2.250485     -0.454521     -0.235839
O            -3.087474      0.055582     -0.942644
C            -0.641861      1.545117      1.140245
H            -0.002110      2.382825      1.409094
H            -1.663834      1.908198      1.038223
H            -0.610718      0.814107      1.945976
C            -0.291509      1.964074     -1.309620
H            -1.343794      2.193192     -1.465768
H             0.243558      2.877051     -1.059783
H             0.120050      1.567476     -2.236296
H             0.011074     -2.440245     -0.195858
H            -0.682013     -0.561924     -1.597121
O            -2.568461     -1.093143      0.914813
H            -3.531417     -1.028490      1.004241
```

k

31
Enegy: -538.08261683

N	8.063114	8.927242	7.235577
C	7.220309	10.137056	7.308630
N	7.928800	11.010567	6.361109
C	9.346560	10.642029	6.397262
C	9.298311	9.106076	6.449182
O	7.691495	7.807710	7.709805
C	9.110562	8.443589	5.083475
C	10.476647	8.495102	7.192216
H	8.315311	8.929925	4.522925
H	8.844220	7.399205	5.234076
H	10.029601	8.484822	4.500858
H	11.398200	8.660858	6.635283
H	10.328074	7.423942	7.306799
H	10.582588	8.934388	8.182699
C	5.813047	9.824848	6.822672
C	7.202109	10.620028	8.764002
H	5.846570	9.456714	5.800579
H	5.195690	10.721357	6.854534
H	5.362186	9.068386	7.460722
H	8.195725	10.917052	9.095539
H	6.860647	9.803808	9.396919
H	6.524132	11.462460	8.888863
C	10.132434	11.222544	5.235113
H	9.642428	11.008061	4.288015
H	11.137322	10.802279	5.213148
H	10.235541	12.301231	5.332287
C	7.667147	12.429413	6.512378
H	8.117429	12.861766	7.416596
H	6.593975	12.603719	6.547690
H	8.055530	12.965985	5.650492
H	9.814462	10.988578	7.334265

l

28
Enegy: -611.62488161

O	-2.786272	1.039016	-0.197765
O	2.536018	1.120380	0.529481
N	-1.680721	0.414981	-0.151131
C	-0.376095	1.110077	0.008478
C	0.546275	-0.086235	0.064283
C	-0.127387	-1.241390	0.070103
C	-1.612539	-1.057419	-0.010377
C	-0.388730	1.925090	1.304271
C	-0.126258	2.011064	-1.198934
C	-2.231422	-1.722228	-1.240644
C	-2.335083	-1.498871	1.265529
C	2.020703	0.080127	0.144613
H	0.311535	-2.225819	0.152625
H	-1.204193	2.644254	1.257445
H	-0.543849	1.278081	2.166053
H	0.557374	2.444118	1.423504
H	-0.107439	1.429342	-2.119387
H	-0.926931	2.744344	-1.269397
H	0.823715	2.527473	-1.084155
H	-3.273729	-1.421281	-1.324962
H	-1.708382	-1.421822	-2.146486
H	-2.185804	-2.806861	-1.149877
H	-1.887725	-1.038480	2.143963
H	-3.379481	-1.199162	1.207983
H	-2.284175	-2.581594	1.373889
N	2.764933	-1.010643	-0.210453
H	2.366371	-1.742193	-0.769116
H	3.762097	-0.883568	-0.241359

m

```
30
Enegy:    -670.77665600
O              2.812328       1.054963       0.000001
O             -2.571713       1.130138      -0.000000
N              1.706945       0.429142      -0.000001
O             -2.624411      -1.124481       0.000000
C              0.392456       1.121366      -0.000000
C             -0.523737      -0.079626      -0.000000
C              0.146192      -1.236598      -0.000000
C              1.631230      -1.049315      -0.000000
C              0.268076       1.979709      -1.259314
C              0.268078       1.979708       1.259314
C              2.299490      -1.605634       1.259667
C              2.299491      -1.605636      -1.259666
C             -1.990229       0.065539      -0.000000
C             -4.059375      -1.045833      -0.000000
H             -0.313383      -2.213138       0.000000
H              1.075545       2.708935      -1.272606
H              0.341110       1.364533      -2.154806
H             -0.687523       2.497395      -1.262657
H              0.341112       1.364531       2.154806
H              1.075546       2.708934       1.272606
H             -0.687522       2.497394       1.262659
H              3.344809      -1.303748       1.273224
H              1.814429      -1.223928       2.155844
H              2.246196      -2.693453       1.267884
H              1.814429      -1.223932      -2.155844
H              3.344809      -1.303749      -1.273223
H              2.246196      -2.693455      -1.267881
H             -4.409709      -0.522224      -0.885324
H             -4.404631      -2.072914      -0.000001
H             -4.409709      -0.522225       0.885324
```

n

```
30
Enegy:   -596.54866389
N              -0.000000      -0.000000       1.512228
O              -0.000000      -0.000000       2.783023
C               0.000000       1.256226       0.720947
C               0.000000       0.696814      -0.676118
C              -0.000000      -0.696814      -0.676118
C              -0.000000      -1.256226       0.720947
C              -1.258929       2.061606       1.045343
H              -1.273853       2.302431       2.106425
H              -1.269467       2.988167       0.472934
H              -2.154136       1.493077       0.801023
C               1.258929       2.061606       1.045343
H               1.269467       2.988167       0.472934
H               1.273853       2.302431       2.106425
H               2.154136       1.493077       0.801023
C               0.000000       1.402304      -1.872511
H               0.000000       2.484041      -1.876985
C               0.000000       0.697195      -3.073348
H               0.000000       1.233578      -4.011502
C              -0.000000      -0.697195      -3.073348
H              -0.000000      -1.233578      -4.011502
C              -0.000000      -1.402304      -1.872511
H              -0.000000      -2.484041      -1.876985
C               1.258929      -2.061606       1.045343
H               1.273853      -2.302431       2.106425
H               1.269467      -2.988167       0.472934
H               2.154136      -1.493077       0.801023
C              -1.258929      -2.061606       1.045343
H              -1.269467      -2.988167       0.472934
H              -1.273853      -2.302431       2.106425
H              -2.154136      -1.493077       0.801023
```

O

```
33
Enegy:   -785.08776864
C          -2.253211      -0.267680       0.000001
C          -1.853455      -1.607894       0.000001
H          -2.615260      -2.372921       0.000001
C          -0.505296      -1.938567       0.000001
H          -0.201600      -2.976343       0.000001
C           0.440251      -0.919456       0.000001
C           0.043271       0.417194       0.000001
C          -1.300839       0.754756       0.000002
H          -1.619853       1.786263       0.000002
C           1.937598      -1.057022      -0.000000
C           2.475408      -1.737822      -1.259650
H           3.560819      -1.663856      -1.275109
H           2.193063      -2.789581      -1.268574
H           2.078164      -1.263817      -2.155074
C           2.475410      -1.737823       1.259648
H           2.078167      -1.263819       2.155073
H           3.560821      -1.663857       1.275105
H           2.193065      -2.789583       1.268571
C           1.222359       1.352285       0.000001
C           1.301700       2.216098      -1.259312
H           0.488133       2.940067      -1.269616
H           2.249395       2.750437      -1.274023
H           1.230753       1.601639      -2.154713
C           1.301701       2.216097       1.259314
H           0.488134       2.940066       1.269619
H           2.249397       2.750436       1.274024
H           1.230755       1.601639       2.154715
C          -3.706603       0.020123       0.000000
O          -3.982142       1.348752      -0.000002
H          -4.947479       1.421012      -0.000004
O          -4.580606      -0.816844      -0.000001
N           2.338184       0.372642       0.000000
O           3.556057       0.733341      -0.000002
```

p

30			
Enegy:	-783.61897073		
C	3.000248	-0.858326	0.112410
C	1.626258	-0.700087	-0.082651
C	1.040802	0.550896	-0.241604
C	1.892658	1.645618	-0.210154
C	3.275310	1.504244	-0.020596
C	3.839638	0.247319	0.147857
C	3.288565	-2.287379	0.292641
C	1.956705	-3.050796	0.066369
H	-0.024352	0.648282	-0.381298
H	1.479121	2.636521	-0.334150
H	3.900612	2.384522	-0.003145
H	4.900671	0.116766	0.304019
N	0.988771	-1.934575	-0.071299
O	4.342495	-2.811316	0.580400
O	-0.269525	-2.096305	-0.126528
C	1.589501	-3.903767	1.276039
H	0.622383	-4.371145	1.110587
H	2.344692	-4.671313	1.418812
H	1.540731	-3.290987	2.175027
C	2.053643	-3.857210	-1.220684
C	2.992175	-4.890306	-1.295902
C	1.250970	-3.586146	-2.328298
C	3.118431	-5.641839	-2.457225
H	3.639487	-5.090825	-0.455410
C	1.380811	-4.342953	-3.489565
H	0.511022	-2.802422	-2.286109
C	2.311585	-5.372232	-3.558806
H	3.850754	-6.435388	-2.501523
H	0.747947	-4.125303	-4.338156
H	2.409250	-5.958426	-4.461313
